# Analysis of bacterial community structure of Fuzhuan tea with different processing techniques

**DOI:** 10.1515/biol-2022-0573

**Published:** 2023-02-09

**Authors:** Shiquan Liu, Taotao Li, Songlin Yu, Xiaohong Zhou, Zhanjun Liu, Xuemao Zhang, Hongmei Cai, Zhiyuan Hu

**Affiliations:** Department of Hunan Provincial Key Lab of Dark Tea and Jin-hua, Hunan City University, Yiyang City, Hunan Province, 413000, China; Yiyang Guan-Longyu Dark Tea Development Co., Ltd, Yiyang City, Hunan Province, 413000, China; Department of Hunan Provincial Key Lab of Dark Tea and Jin-hua, Hunan City University, No. 518 Yingbin Road, Yiyang City, Hunan Province, 413000, China

**Keywords:** Fuzhuan tea, processing technique, high-throughput sequencing, bacterial community structure

## Abstract

The composition and diversity of microbial communities are of considerable significance to the quality development of *Camellia sinensis* (Fuzhuan tea). In this study, we examined differences in the bacterial community structures of loose, lightly-pressed, hand-made, and machine-pressed Fuzhuan teas and raw dark tea. We observed notable differences in the bacterial communities of the five groups, where there were only 51 consensus sequences. ASV/OTU Venn diagram, Chao1, Ace, Simpson indices, and dilution curve analyses consistently revealed that machine-pressed tea exhibited the highest bacterial diversity. Taxonomically, *Actinobacteria*, *Firmicutes*, *Proteobacteria*, and *Cyanobacteria* were the dominant bacterial phyla in each group, whereas *Corynebacterium*, *Methylobacterium*, and *Bifidobacterium* were the dominant genera. Our findings revealed significant differences in the bacterial community structures of different Fuzhuan tea products derived from the same raw material, with bacterial diversity rising with increased product compaction.

## 1 Introduction

Native to the Hunan province of China, Fuzhuan tea is traditionally produced via fungal fermentation of *Camellia sinensis* L. (raw dark tea) leaves and is processed through blending, screening, stacking, steam pressing, fermentation, drying, and packaging [1]. This tea has been demonstrated to have unique physiological benefits, such as conditioning of intestines [2,3] and antibacterial [4], blood lipid-lowering [5], liver protection [6], and immunoregulatory [7,8] functions, and presents great potential for product development.

Fungal fermentation during the processing of Fuzhuan tea is considered to be the key factor contributing to its unique flavour and properties [9]. In recent years, advances in molecular biology techniques have allowed for a better understanding of the microbial diversity in Fuzhuan tea. To date, research on microorganisms in Fuzhuan tea has mainly focused on the isolation and identification of dominant strains [10], the physiological activity of their metabolites [11], and structural changes in microbial flora during the production process. With the development and application of high-throughput sequencing technology, an increased number of studies have applied Illumina MiSeq sequencing to study the structures of microbial communities in Fuzhuan tea. Although it is well known that the microbial community structure exerts a pronounced influence on tea quality, it remains unclear how different processing techniques control the microbial community structure in Fuzhuan tea.

The rapid development of the Fuzhuan tea industry has advanced its processing technology and facilities, superseding the traditional fermentation technology with modern fermentation. Also, different types of processing technology have evolved such as bulk, lightly pressed, hand-made, and machine-pressed Fuzhuan tea. Naturally, these processing techniques contribute to creating different microbial communities in Fuzhuan tea which in turn influences its quality attributes [12]. Consequently, analysis of microbial communities can provide actionable insights into the drivers of tea quality attributes. With the advance in high-throughput sequencing technology, sequencing results can reliably and comprehensively reflect the community structures of the sample microbial populations. In this study, using Illumina MiSeq sequencing, we analysed the structures of the bacterial communities associated with raw dark tea material and loose, lightly-pressed, hand-made, and machine-pressed Fuzhuan teas. The correlation between the processing technology and the Fuzhuan tea microbial community can provide new and actionable insights into the regulation of its fermentation as well as the enrichment and improvement of its processing.

## 2 Materials and methods

### 2.1 Tea samples

Tea samples were collected from a production workshop of Yiyang Guan-Longyu Dark Tea Development Co., Ltd, Hunan Province, China, on 5 March 2021. The samples included raw dark tea (G0), loose Fuzhuan tea (G1), lightly-pressed Fuzhuan tea (G2), hand-made Fuzhuan tea (G3), and machine-pressed Fuzhuan tea (G4) and were derived from the same batch of raw dark tea material. Among these, G1 was loose tea, while G2, G3, and G4 were pressed teas, with the pressing degree gradually rising from G2 to G4. All the samples were analysed in three biological replicates.

### 2.2 PCR amplification

Genomic DNA was extracted from the samples, using the Soil DNA kit (OMEGA BioTek, USA), with the purity and concentration of the extracted DNA determined using agarose gel electrophoresis. Aliquots of the purified DNA were diluted to a concentration of 1 ng/μL with sterile water and stored at −80°C for later use [13]. The total DNA of the samples was quantified using a NanoDrop 2000 ultramicroprotein analyser (Thermo Fisher Scientific, USA). The DNA mass concentration and the OD 260/OD 280 ratio were read for comparative analysis. The determination of DNA purity was based on the OD 260/OD 280 ratio, which ranged from 1.6 to 1.8 for highly purified DNA. The values below and above this range indicated excessive protein content and RNA, respectively, in the sample.

Using DNA extracted from G0, G1, G2, G3, and G4 samples as templates, we used the primer pair 338F (5′-TCCGTAGGTGAACCTGCGG-3′) and 806R (5′-GGACTACHVGGGTWTCTAAT-3′) to amplify the V3–V4 variable regions of bacterial 16S rRNA gene sequences.

The PCR reaction mixtures contained 10 ng of DNA template, 2.5 μM upstream and downstream primers, and 2.5 μM DNA polymerase (Hieff® Taq DNA Polymerase, 5 U/μL), made up to a final volume of 30 μL with PCR buffer. The PCR amplification conditions were as follows: pre-denaturation at 98°C for 60 s, followed by 30 cycles of denaturation at 98°C for 10 s, annealing at 50°C for 30 s, and extension at 72°C for 30 s, with a final extension at 72°C for 5 min. PCR products were detected using 2% agarose gel electrophoresis. The samples were mixed at equal concentrations according to the PCR product concentration, and the PCR products were detected using 2% agarose gel electrophoresis after thorough mixing.

### 2.3 Library construction and sequencing

Having qualified the constructed library based on Qubit quantification and library detection, we obtained an original data file by using the Illumina (MiSeq pe300) platform for sequencing, with the data being converted to sequenced readings after base calling. The results were stored in FASTQ format, containing information on readings and corresponding sequencing quality.

### 2.4 Bioinformatics analysis

The original data were spliced and filtered to obtain clean data. Based on the valid data, we performed sequence denoising or operational taxonomic unit (OTU) clustering, using the process of QIIME2 dada2 analysis [14] or Vsearch analysis [15], followed by species classification analysis. Based on denoising or clustering results, each sequence was annotated to obtain the corresponding species information and species-based abundance distributions.

Amplicon sequence variants (ASVs) or OTUs were analysed to obtain information on within-sample species richness and evenness [16]. Multi-sequence alignment of the ASVs or OTUs was performed, based on which, we constructed phylogenetic trees. Performing dimensionality reduction analysis, such as principal coordinates analysis (PCoA) and principal component analysis, we examined differences in the community structures of the different samples or groups [17].

The number of ASVs/OTUs per sample was obtained using QIIME2, and the numbers of common and unique ASVs/OTUs among the samples were displayed using Venn diagrams [18]. The sample alpha diversity index was assessed using the diversity alpha-rarefaction command of QIIME2 software [19].

## 3 Results and discussion

### 3.1 Sequencing data statistics

The number of valid tags in the five groups of the samples was more than 53,000, with the Q20 and Q30 values greater than 95.54 and 88.58, respectively. More than 29,000 high-quality sequences were obtained. For each sample, we obtained more than 270 ASV/OTU sequences, with an average data quality of 35 or higher. For all the samples, the sequencing quality met the requirements for subsequent analysis.

### 3.2 ASV/OTU-Venn statistics and classification analysis

**Figure 1:**
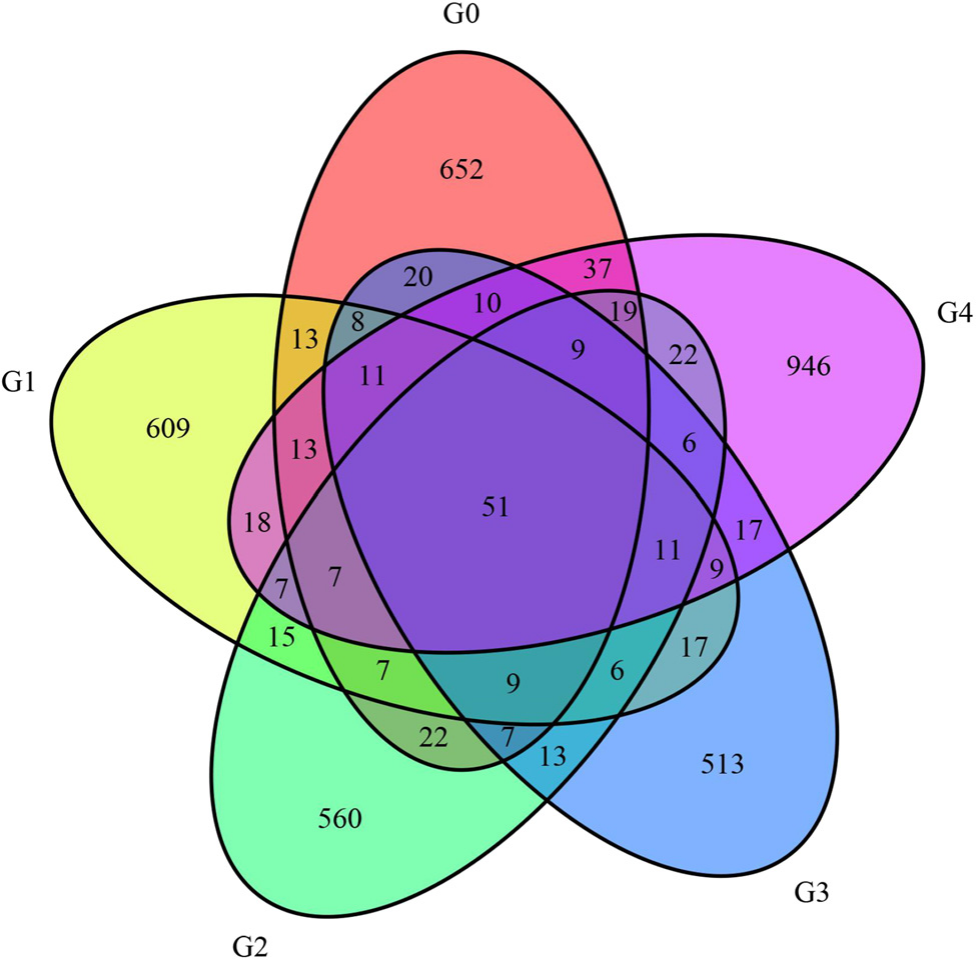
ASV/OTU Venn map. Note: Overlap area: the number of ASV/OTU shared between different samples. Non-overlapping areas: the number of ASV/OTU specific of each sample.

The Venn map was constructed to depict the similarity in the bacterial populations of the different samples (Figure 1). Among the five assessed tea groups, the lowest and highest numbers of unique sequences were detected in G3 (513) and G4 (946), respectively. We detected only 51 consensus sequences among the five groups. This in turn indicated that the different processing techniques exert a significant influence on the microbial community structure in Fuzhuan tea. Group G4 with the highest number of unique sequences may be attributable to the destructive nature of machine pressing as this process may lead to the pronounced releases of contents from disrupted tea tissues which may be conducive to the growth of different bacteria.

### 3.3 Alpha diversity index analysis

**Figure 2:**
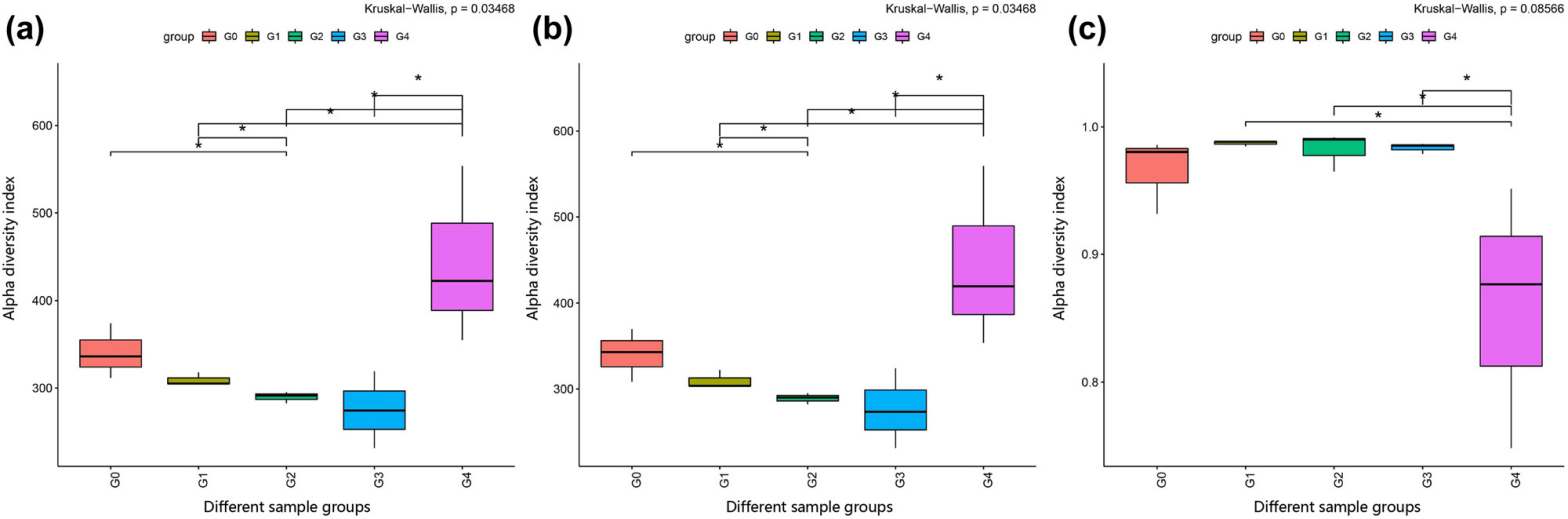
Alpha diversity index (a) ACE index, (b) Chao1 index, and (c) Simpson index. Note: The horizontal lines in the figure represent the two groups with differences (*p* < 0.05) and are marked with *.

Values obtained for the Alpha diversity Chao1, Ace, and Simpson indices of each sample are shown in Table 1 and Figure 2. These results showed that the Chao1 and Ace indices of the samples from the five groups varied between 274 and 444, with the highest values obtained for group G4. We detected significant differences between groups G0 and G2, G1 and G2, G1 and G4, G2 and G4, and G3 and G4. Group G4 was found to significantly differ from all the other groups, thus indicating that the machine pressing process is conducive to microbial growth and higher bacterial abundance.

**Table 1:** Statistics of alpha diversity index (1, 2, and 3 after the sample numbers represent three parallel assays)

Sample name	Single sample ACE index	Between-group mean	Single sample Chao1 index	Between-group mean	Single sample Simpson index	Between-group mean
G0.1	373.9009	340.6048	369.5882	340.3865	0.9318	0.9660
G0.2	311.7155		308.5714		0.9803	
G0.3	336.1981		343.0000		0.9859	
G1.1	317.9485	309.0535	322.1667	309.6984	0.9846	0.9873
G1.2	305.1103		303.5000		0.9889	
G1.3	304.1018		303.4286		0.9883	
G2.1	291.3519	289.7892	289.9091	289.0067	0.9650	0.9823
G2.2	295.3543		295.0000		0.9919	
G2.3	282.6615		282.1111		0.9901	
G3.1	231.3358	274.8899	231.1429	276.2322	0.9867	0.9836
G3.2	319.1115		324.1250		0.9788	
G3.3	274.2225		273.4286		0.9852	
G4.1	553.7025	443.7158	559.4000	444.2127	0.7483	0.8589
G4.2	354.8699		353.5714		0.9515	
G4.3	422.5749		419.6667		0.8768	

The Simpson index is mainly used as a measure of species diversity. The lower the Simpson index value is, the higher the species diversity of a sample is. As shown in Table 1, the Simpson index values of the five groups ranged from 0.8589 to 0.9873, with the lowest value for group G4, thus indicating that the bacterial community in the machine-pressed tea is characterized by the highest bacterial diversity. We detected significant differences between group G4 and the other three processed teas with respect to the Simpson index, again indicating that the machine-pressed tea led to the highest bacterial diversity, as was consistent with the results from the ASV/OTU-Venn diagram analysis.

### 3.4 Rarefaction curve

**Figure 3:**
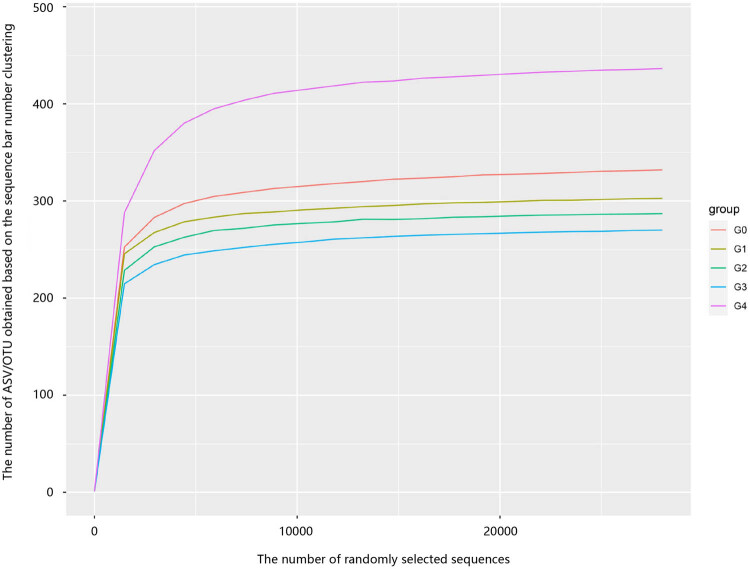
Curve of dilution.

Figure 3 shows the sample dilution curve. The 15 samples of the five groups were all within 2,500 sequences. With the increased number of sequencing samples, the curve showed a sharp rise, indicating that a large number of species was found in the sample community. When the number of sequences exceeded 2,500, the curve flattened, indicating that the number of species in this sample did not rise with the increased number of sequences. The number of samples sequenced was more than 20,000, indicating that the sequencing volume of each sample was sufficient. From the perspective of the number of new ASVs, compared to group G0, we detected a reduction in the final number of new ASVs in G1, G2, and G3 and an increase in the final number of new ASVs in G4. Therefore, these findings indicate that the processing technique exerts a significant impact on the growth of bacteria and that the machine pressing process was more conducive to increasing bacterial populations than the other processes.

### 3.5 PCoA analysis

**Figure 4:**
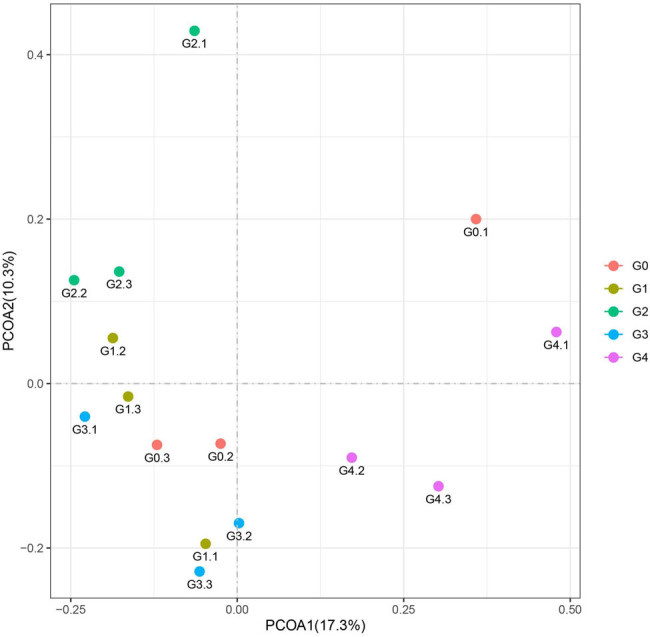
PCoA analysis. Note: 1, 2, and 3 after the sample numbers represent three parallel assays.

**Figure 5:**
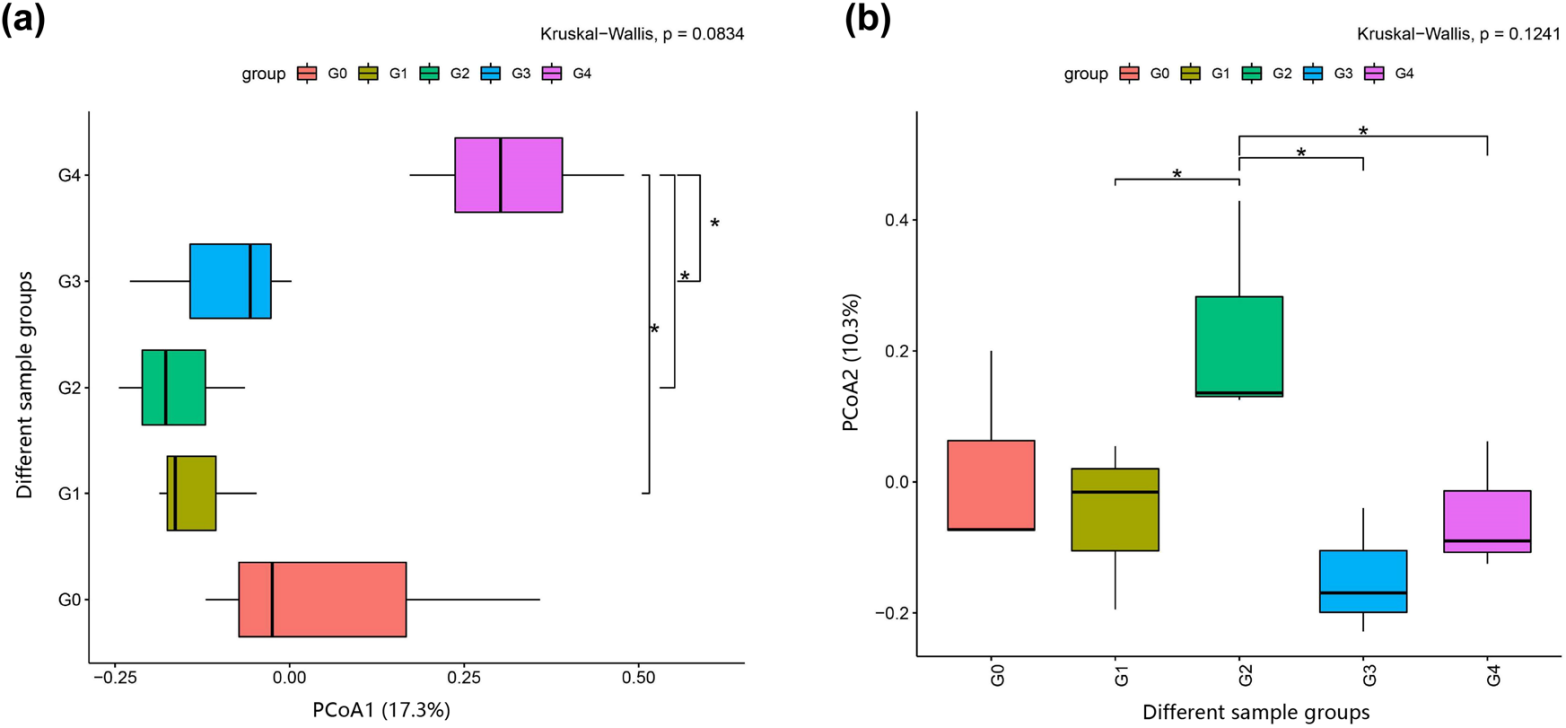
PCoA principal component analysis: (a) first principal component and (b) second principal component. Note: The horizontal lines in the figure represent the two groups with differences (*p* < 0.05) and are marked with *.

The results of PCoA analyses of the five groups are shown in Figure 4. The larger distance between G4 and G0 than between the other groups showed the distinct difference in the species diversity of the G4 bacterial community. As can be seen in Figure 5, according to principal component 1, G4 was significantly different from G1, G2, and G3 (Figure 5a). However, according to principal component 2, G1 was significantly different from G2, while G2 was significantly different from G3 and G4 (Figure 5b). Therefore, these findings indicate that the different processing techniques significantly influence the compositions of the bacterial communities in the samples. The composition of the bacterial community in G4 was affected by the processing and differed from G1, G2, and G3 significantly. This was consistent with the findings of the ASV/OTU-Venn diagram, Chao1, Ace, Simpson indices, and dilution curve analyses.

### 3.6 Species annotation analysis

The results of the taxonomic annotations are shown in Table 2. The highest number of annotated ASV/OTU sequences in the five groups was seven phyla, 23 classes, 41 orders, 154 families, 532 genera, and 231 species, with significant differences identified among the groups. Compared to the other groups, the number of ASV/OTU sequences annotated to each level in G4 was highest, indicating that the machine pressing process most significantly affected the composition of the bacterial communities in Fuzhuan tea, as was also consistent with the previous analysis results.

**Table 2:** Statistical table of species of tea samples from annotations to grades (ASV/OTU sequence)

Group name	Kingdom	Phylum	Class	Order	Family	Genus	Species	Unknow
G0	90	4	14	36	114	411	134	92
G1	67	8	20	27	95	374	141	79
G2	65	5	13	32	87	356	134	79
G3	68	4	12	31	79	336	116	71
G4	97	7	23	41	154	532	231	108

At the phylum level of classification, we annotated 17 phyla with clear status among the Fuzhuan tea samples subjected to the different processing techniques; namely, *Actinobacteria*, *Firmicutes*, *Proteobacteria*, *Cyanobacteria*, *Bacteroidetes*, *Verrucomicrobia*, *Fusobacteria*, *Synergistetes*, *Deinococcus-Thermus*, *Planctomycetes*, *Patescibacteria*, *Tenericutes*, *Chloroflexi*, *Spirochaetes*, *Nitrospirae*, *Gemmatimonadetes*, and *Acidobacteria*, the top ten of which are shown in Table 3. Among the five groups, *Actinobacteria* was identified as the predominant bacterial phylum, followed by *Firmicutes*, *Proteobacteria*, and *Cyanobacteria*. This was consistent with the results of MiSeq sequencing analysis of dark tea products, such as Fuzhuan tea and pu-erh tea, reported by Fu et al. [20], who found *Firmicutes* and *Actinobacteria* to be the dominant phyla.

**Table 3:** Species statistics at the phylum level (top ten in abundance)

Serial number	Microbial species	G0	G1	G2	G3	G4
1	*Actinobacteria*	24,245	28,214	26,254	26,914	17,256
2	*Firmicutes*	18,645	19,714	21,442	19,357	15,103
3	*Proteobacteria*	12,397	9,691	13,882	8,995	8,528
4	*Cyanobacteria*	12,062	6,646	2,363	8,485	29,915
5	*Bacteroidetes*	7,919	9,985	11,173	8,485	5,487
6	Unknown	4,611	4,642	4,701	4,614	4,034
7	*Verrucomicrobia*	1,433	919	1,017	1,467	823
8	*Fusobacteria*	1,041	1,379	976	1,322	656
9	*Synergistetes*	987	133	413	755	339
10	*Deinococcus-Thermus*	341	491	243	1,277	339

With respect to the top five predominant phyla, the numbers of *Actinobacteria*, *Firmicutes*, and *Bacteroidetes* in G1 rose by varying degrees when compared to those in G0. The numbers of *Proteobacteria* and *Cyanobacteria* fell in G1 compared to those in G0. Compared to those in G0, the numbers of *Actinobacteria*, *Firmicutes*, *Proteobacteria*, and *Bacteroidetes* in G2 increased by a different degree, whereas there was a significant reduction in the number of *Cyanobacteria*. The pattern of phyla was found to be essentially the same between G3 and G1. Only the number of *Cyanobacteria* significantly increased (1.48 times), while the numbers of *Actinobacteria*, *Firmicutes*, *Proteobacteria*, and *Bacteroidetes* decreased in G4 when compared to G0. These observations verified that the different processing techniques significantly influenced the structures of the bacterial communities in Fuzhuan tea.

At the genus level, we annotated 532 genera with clear status in the different samples, among which, *Corynebacterium*, *Methylobacterium*, *Bifidobacterium*, *Faecalibacterium*, *Escherichia–Shigella*, *Actinomyces*, *Bacteroides*, *Collinsella*, *Sphingomonas*, *Prevotella*, *Candidatu*s *Xiphinematobacter*, *Caldicoprobacter*, *Aminobacterium*, uncultured *Acidothermaceae*, *Leptotrichia*, *Coprostanoligenes* group, and *Veillonella* were detected commonly. The number of species in each group at the genus level is shown in Table 4. Among the five groups, *Corynebacterium* was most predominant, followed by *Methylobacterium* and *Bifidobacterium*. Compared to G0, a varying degree of increased numbers of *Corynebacterium*, *Faecalibacterium*, *Escherichia–Shigella*, *Actinomyces*, and *Collinsella* was detected in G1, whereas a reduction was observed for all the other genera, among which, a significant (approximately 10%) reduction in the number of *Methylobacterium* was found. Increases to a different extent in the numbers of *Corynebacterium*, *Faecalibacterium*, *Escherichia–Shigella*, and *Bacteroides* were observed for G2 when compared to G0, whereas the numbers of all the other genera declined during the processing. The varying degrees of increases in the numbers of *Corynebacterium*, *Faecalibacterium*, and *Sphingomonas* were found in G3 when compared to G0, with the numbers of the remaining genera declining, among which the number of *Methylobacterium* significantly fell by approximately 10%. Only the number of *Collinsella* increased in G4 compared to G0, with the numbers of all the other genera decreasing, in particular, the numbers of the unidentified genera. Collectively, these findings highlight the significant effects of the Fuzhuan tea processing techniques on the bacterial community structure, with important implications for the final tea products.

**Table 4:** Species statistics at the genus level (top ten in abundance)

Serial number	Microbial species	G0	G1	G2	G3	G4
1	Unknown	23,620	19,111	14,942	20,147	40,309
2	*Corynebacterium*	12,372	15,617	13,518	16,290	8,057
3	*Methylobacterium*	4,476	530	4,909	428	1,892
4	*Bifidobacterium*	3,033	1,604	2,882	2,030	2,350
5	*Faecalibacterium*	2,425	3,370	4,493	4,047	2,457
6	*Escherichia–Shigella*	2,078	2,427	2,141	1,777	1,382
7	*Actinomyces*	1,837	1,985	1,809	1,544	1,329
8	*Bacteroides*	1,677	1,488	2,080	826	1,106
9	*Collinsella*	1,380	2,205	1,116	832	1,400
10	*Sphingomonas*	1,336	1,272	1,169	1,421	651

**Figure 6:**
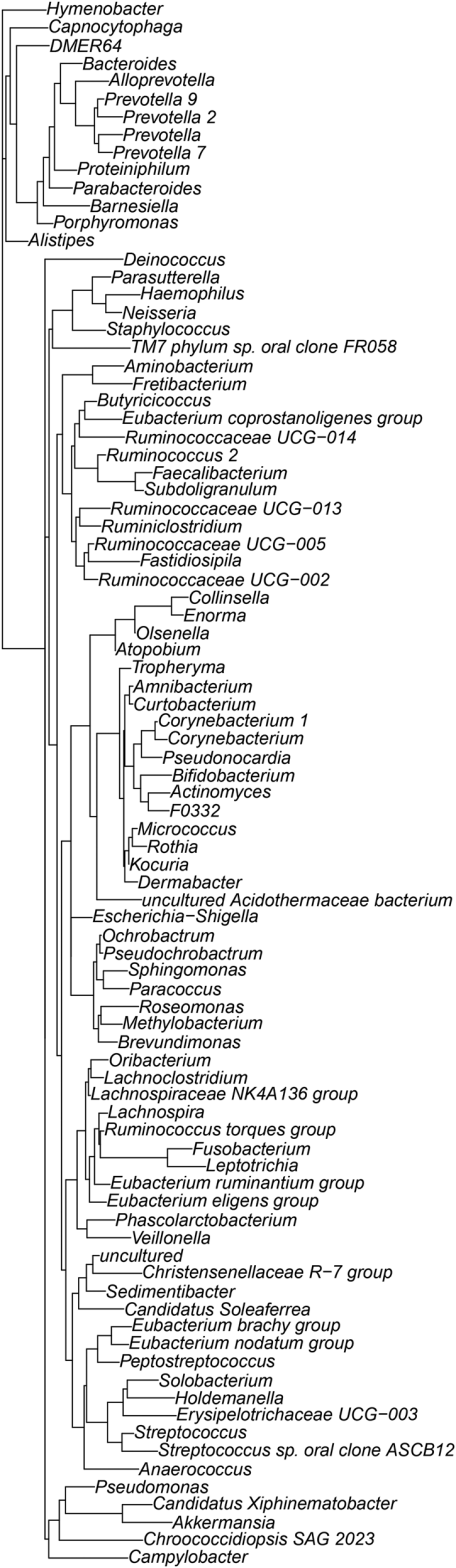
Phylogenetic tree of top 100 genera in different sample groups.

To further study the phylogenetic relationship among the species at the genus level, we used MEGA 5 to obtain representative sequences of the top 100 genera based on multiple sequence alignment [21]. The results shown in Figure 6 point to the extremely rich bacterial diversity among the differently processed Fuzhuan teas. However, the relationships among these microbial populations and their interaction effects on the product quality need to be further assessed.

## 4 Conclusion

The flavour, colour, and aroma attributes of fermented teas and control over them primarily depend on the activities of microorganisms associated with the fermentation process. A better understanding of the composition of this microbial community is required to improve tea product quality [22]. Previous studies have shown that *Klebsiella*, *Lactococcus*, and *Bacillus* play a leading role in the fermentation process of Fuzhuan tea as well as participate in the generation of a large number of flavour compounds in the tea [23]. *Aspergillus*, *Candida*, *Debaryomyces*, *Penicillium*, and *Saccharomycetales* significantly contribute to the formation of the unique aroma of Fuzhuan tea [24]. *Aspergillus niger*, *Aspergillus pallidofulvus*, *Aspergillus sesamicola*, and *Penicillium mangini* can transform theophylline into theophylline in pu-erh tea, thus affecting its quality [25]. Chen et al. [26] reported the dependency of the metabolites of Fuzhuan tea on different production regions and attributed the difference in the tea microbial community to this.

In this study, using Illumina MiSeq technology, the bacterial structure was analysed in the finished products of the differently processed Fuzhuan teas. Seven phyla, 23 classes, 41 orders, 154 families, 532 genera, and 231 species were detected. Among the five groups analysed, only 51 consensus sequences were identified. Among the four groups (G1, G1, G3, and G4), G3 and G4 had the lowest (513) and highest (946) numbers of unique sequences, respectively. Alpha diversity index, dilution curve, rank abundance curve, and PCoA analyses consistently pointed to the significant influence of the different processing techniques on the structure of the bacterial communities in the finished Fuzhuan tea products, with the machine-pressed tea (G4) having the highest bacterial species abundance and diversity.

We also detected significant differences among the different tea products with respect to the number of ASV/OTU sequences annotated at the levels of kingdom, phylum, class, order, family, genus, and species. G4 had the highest number of ASV/OTU sequences annotated to each level. *Actinobacteria* dominated the phyla level, followed by *Firmicutes*, *Proteobacteria*, and *Cyanobacteria*, whereas the genera were dominated by *Corynebacterium*, followed by *Methylobacterium* and *Bifidobacterium*. Some of these bacteria, such as *Methylobacterium* and *Bifidobacterium*, were loosely associated with the quality of fermented tea [27]. The presence of *Faecalibacterium* in tea was associated with the regulation of the intestinal tract [28]. Xynanase secreted by *Actinobacteria* helps to improve the quality of beverages and baked goods [29]. Amino acids and small molecules produced by *Corynebacterium* can enhance the concentration and taste of tea soup [30].

In the present study, the differences in the bacterial community structure among the different types of Fuzhuan tea were attributed mainly to the differences in the compression degree of the raw materials during the processing. Unlike loose Fuzhuan tea, not pressed, lightly-pressed, hand-made, and machine-pressed Fuzhuan teas, pressed with a progressive degree, damaged the tea tissues to a different extent, thus changing the releases of the contents of the leaf cells. This in turn plays an important role in influencing the subsequent fermentation. The results consistently indicated that the machine-pressed Fuzhuan tea contained the highest abundance and diversity of bacterial species. In other words, the excessive releases of intracellular tea contents significantly promoted the growth and development of bacteria. The degree of compaction among the four processed teas progressively rose from G1 to G4. Given the difference in compactness, the differently processed teas were characterized by different internal air circulation and humidity which in turn affected the growth of bacteria, and thus, the bacterial community structure. Overall, the change in the bacterial community structure during the fermentation of Fuzhuan tea significantly depended on the product morphology.

In this study, Illumina MiSeq sequencing was used to provide insights into the structure and diversity of the bacterial communities of the differently processed Fuzhuan teas. However, the complex nature of these tea communities did not allow for the annotation of all the detected ASV/OTU sequences to specific species, which warrants further analyses. An additional focus in future is required on the specific metabolic mechanisms of bacterial community members with respect to the development of the quality attributes of Fuzhuan tea, such as their respective contributions to flavour formation. Given these insights, it may be feasible to control the different processing conditions to fully exploit the biotransformation properties of the tea microbiota. This in turn may enhance the quality of Fuzhuan tea, and thus, raise its manufacturing technology as well as quality control and assurance management to a new level.
